# Antimicrobial consumption assessment as a part of infection control surveillance exemplified by bloodstream infection treatment in NICUs

**DOI:** 10.1186/2047-2994-4-S1-P182

**Published:** 2015-06-16

**Authors:** A Różańska, J Wójkowska-Mach, M Bulanda, Polish Neonatal Surveillance Network

**Affiliations:** 1 Chair of Microbiology, Jagiellonian University Medical College, Kraków, Poland

## Introduction

Newborns are a population in which antibiotic usage is extremely high.

## Objectives

The aim of this study was an assessment of antibiotic usage in BSI treatment in the Polish Neonatology Surveillance Network (PNSN) and determining the possibility of applying this kind of analysis in infection control.

## Methods

Data were collected between 01.01.2009 and 12.31.2013 in five tertiary academic neonatal intensive care units (NICU) that took part in the PNSN. The PNSN is a prospective national surveillance system for the most relevant infections in the group of very low birth weight (birth weight <1500 g, VLBW) infants in Poland.

Case BSI patients (both: early- and late-onset) were defined according to Gastmeier et al. [1] with modifications.

## Results

The total duration of antibiotic therapy for 773 cases of BSI, which are incorporated into the present analysis, amounted to 14 056 DOTs or 381.6 DDDs.

The median length of antibiotic therapy for a single case of BSI, regardless of microbiological confirmation or its lack, amounted to 8 days; whereas the median consumption expressed in DDD was 0.13. In the case of LC-BSI, median DOT was 8 days, and consumption – 0.12 DDD. Median length of therapy was shorter for unconfirmed cases: 7 days, while the consumption of antibiotics was higher – 0.14 DDD (p<0.0001). High consumption of glycopeptideds expressed in DOTs was observed in studied population.

## Conclusion

Application of classical methods of microbiological diagnostics significantly reduces the consumption of antibiotics expressed by DDD, however, the high consumption of glycopeptides indicates the necessity of applying rapid diagnostic assays.

Nevertheless, the assessment of antibiotic consumption in neonatal units represents a methodological challenge and requires the use of different measurement tools.

Analysis of antibiotic consumption is an essential component of infection control, especially for NICU patients – for effective planning and reliable evaluation of interrelationships between individual elements of control programs.

## Disclosure of interest

None declared.
